# Upregulation of NDRG1 predicts poor outcome and facilitates disease progression by influencing the EMT process in bladder cancer

**DOI:** 10.1038/s41598-019-41660-w

**Published:** 2019-03-26

**Authors:** Aiwei Li, Xi Zhu, Chanjuan Wang, Shuo Yang, Yan Qiao, Rui Qiao, Jie Zhang

**Affiliations:** 1 Department of Laboratory Medicine, Peking University Third Hospital, No.49 North Garden Road, Haidian District, Beijing, 100191 China; 2 Department of Urology, Friendship Hospital Affiliated to Capital Medical University, 95th Yong An Road, Xuan Wu District, Beijing, 100050 China

## Abstract

N-myc downstream regulated gene 1 (NDRG1) is an intracellular protein involved in cell differentiation and was recently reported to exert various effects in several cancers. However, its expression and role in bladder cancer remain unclear. Our study enrolled 100 bladder cancer patients to detect NDRG1 expression in tumour tissues by immunohistochemistry. Correlations between NDRG1 expression and clinical factors were analysed. An NDRG1 overexpression plasmid and NDRG1 siRNAs were transfected into bladder cancer cell lines. Cell biological behaviours were assessed by CCK-8, flow cytometry, wound healing and Transwell assays. Additionally, the influence of NDRG1 on epithelial-mesenchymal transition (EMT) was investigated by western blotting and real-time PCR. NDRG1 expression in urine from bladder cancer patients was examined by ELISA. NDRG1 protein levels were significantly increased in bladder cancer patients and correlated with tumour stage (p = 0.025), lymph node metastasis (p = 0.034) and overall survival (p = 0.016). Patients with high NDRG1 expression had poorer outcomes than those with low NDRG1 expression. NDRG1 overexpression was associated with increased cell proliferation, migration, and invasion and decreased apoptotic cell numbers; NDRG1 knockdown resulted in the inverse effects. Moreover, upregulated NDRG1 expression was associated with downregulated Cytokeratin 7 and Claudin-1 expression and upregulated N-cad, β-catenin and slug expression. Downregulated NDRG1 expression was associated with the inverse effects. Urine protein levels could distinguish bladder cancer patients from healthy controls, with an area under the curve of 0.909. NDRG1 promoted EMT in bladder cancer and could be an effective diagnostic and prognostic biomarker in bladder cancer patients.

## Introduction

Bladder cancer is the most common tumour of the urinary system worldwide, with an estimated 549,000 new cases in 2018^1^. According to the classification, approximately 75% of patients have non-muscle-invasive bladder cancer (NMIBC) initially treated with transurethral resection. However, the recurrence rate exceeds 70% and 10~30% of patients readily progress to muscle-invasive bladder cancer (MIBC), which leads to reduced long-term survival^2^. Cystoscopy is the gold standard for diagnosis and surveillance, but this invasive examination is inconvenient and very painful to patients^3^. Therefore, exploration of the molecular pathogenesis of this disease and identification of effective diagnostic and prognostic biomarkers are urgently needed.

N-myc downstream regulated gene 1 (NDRG1) is the first-discovered member of the NDRG family; mutation of this gene is linked to a disease named hereditary motor and sensory neuropathy-Lom (HMSNL)^4,5^. NDRG1 is involved in many biological processes, including cell differentiation, stress responses, and immunity^6^. Bladder cancer is closely related to smoking and is primarily induced by chemical components that cause DNA damage and hypoxia^7,8^. Some reports have demonstrated that hypoxia-inducible factor 1-alpha (HIF-1α), an indicator of hypoxia, is highly expressed in bladder cancer and that patients with high HIF-1α expression have poor prognoses^7,9^. Moreover, reports indicate that NDRG1 is upregulated by HIF-1 and that the NDRG1 protein level could more accurately reflect tumour hypoxia than that of HIF-1^10,11^, suggesting that NDRG1 may play a role in the development of bladder cancer and is a potential biomarker. In the past decade, interest in NDRG1 as a vital contributor to cancer development has increased^12–16^. However, NDRG1 exerts contradictory effects depending primarily on the tissue type affected; whether it acts as a tumour promoter or suppressor in bladder cancer remains to be further elucidated.

Epithelial-mesenchymal transition (EMT) is a cellular process by which cells lose their epithelial traits and acquire mesenchymal features^17^. EMT is a crucial first step in the development of epithelial-derived malignancies because it improves the ability of tumour cells to migrate and thus invade the surrounding matrix. Cadherin switching, an event involving the loss of E-cadherin (E-cad) expression and the gain of N-cadherin (N-cad) expression, is a unique pattern of EMT and has been shown to be associated with increased cell invasiveness and poor outcome in patients with bladder cancer^18^. Previous investigators have suggested that NDRG1 influences the process of EMT and might be associated with cadherin switching^13,14,19^. Therefore, we sought to investigate the expression and function of NDRG1 in bladder cancer and further reveal the ways in which it is involved in EMT.

## Results

### High NDRG1 mRNA and protein expression in bladder cancer

**Figure 1:**
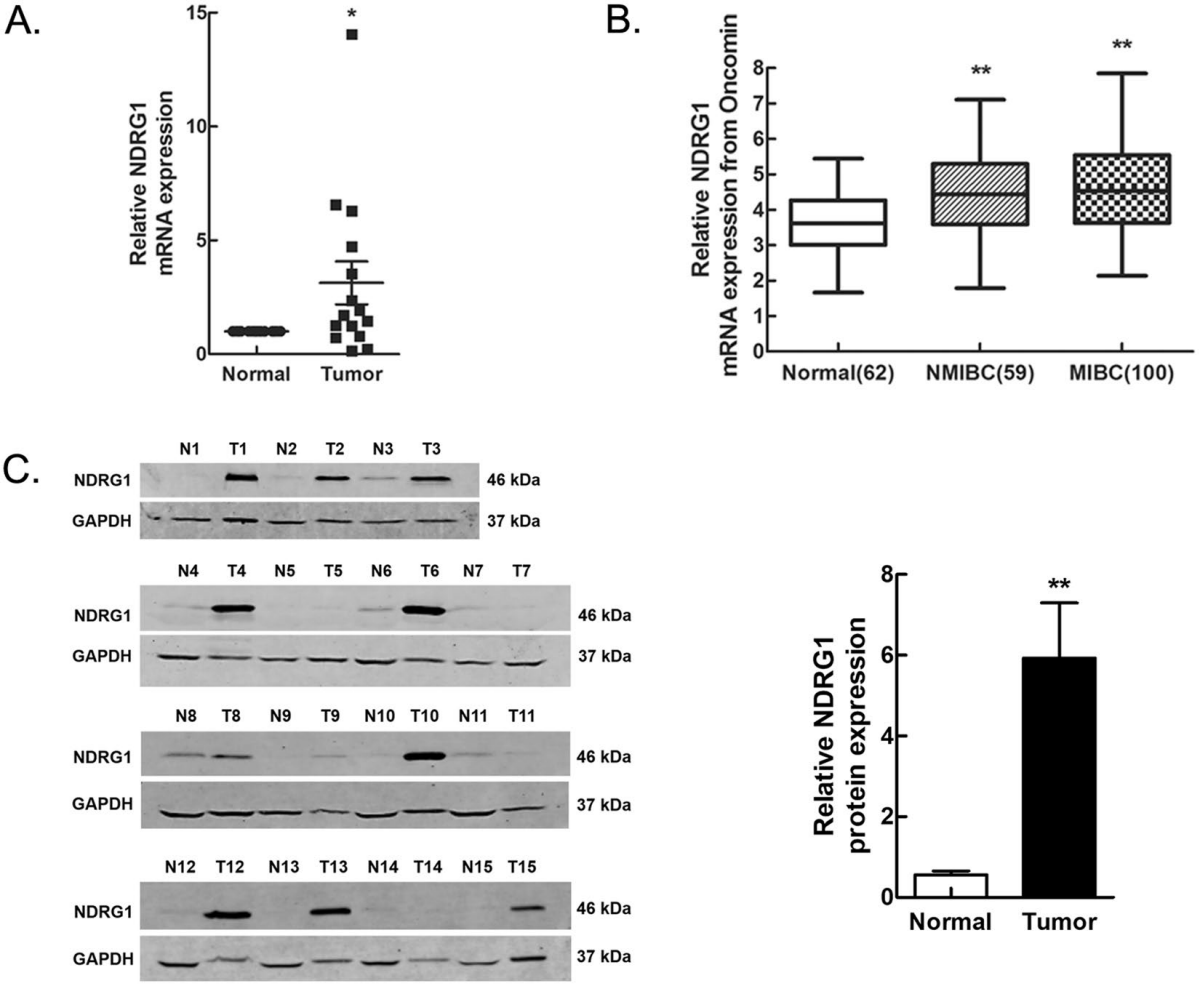
NDRG1 is highly expressed in bladder cancer. (**A**) NDRG1 mRNA and (**C**) protein expression in bladder cancer tissues was examined by real-time PCR and western blot analyses; n = 15, paired t-test, *p < 0.05, **p < 0.01. T: bladder tumour tissue, N: paired tumour-free tissue. (**B**) A meta-analysis of NDRG1 mRNA expression from three Oncomine datasets; LSD test, **p < 0.0001.

**Figure 2:**
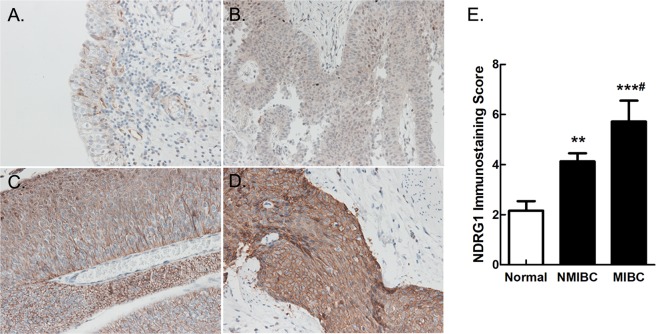
Pathological sections from bladder cancer patients were examined by immunochemistry. (**A**) Low expression of NDRG1 in tumour-free tissue; (**B**) Low expression of NDRG1 in NMIBC tissue; High expression of NDRG1 in (**C**) NMIBC and (**D**) MIBC tissue. NMIBC: non-muscle-invasive bladder cancer, MIBC: muscle-invasive bladder cancer. 200 × magnification. (**E**) The immunostaining score of NDRG1 protein expression in bladder cancer; LSD test, Normal group as the control, **p = 0.00738, ***p = 0.000049. NMIBC group as the control, ^#^p = 0.0211.

To identify the expression of NDRG1 in human tissues, we first obtained 15 pairs of tumour and corresponding tumour-free samples from bladder cancer patients for analysis. The results of the real-time PCR and western blot analyses revealed that the NDRG1 mRNA (p = 0.040) and protein (p = 0.002) expression levels were significantly higher in bladder tumour tissues than in paired tumour-free tissues (Fig. 1A,C). We further validated the high mRNA expression level of NDRG1 (p < 0.0001) in three independent microarray datasets from the Oncomine database^20–22^ but found no significant difference between patients with NMIBC and those with MIBC (p = 0.446) (Fig. 1B). We then analysed the protein expression level of NDRG1 in 100 pathological sections by immunohistochemistry (IHC) (Fig. 2). The immunoscore of the NDRG1 protein in bladder cancer tissues was higher than that in tumour-free tissues (p = 0.000256), and the immunoscore in MIBC patients was significantly higher than that in NMIBC patients (p = 0.0211). In addition, in bladder cancer, NDRG1 was mainly highly expressed in the cytoplasm (62%) and on the cell membrane (38%), but nuclear staining also existed.

### Correlation between NDRG1 expression and clinical factors

The correlation between NDRG1 protein expression and clinical factors was also analysed (Table 1). The median age at diagnosis of the 100 enrolled bladder cancer patients was 68 years (27–88 years). Lymph node metastasis (p = 0.034) and TNM stage (p = 0.025) were associated with high NDRG1 expression. However, age (p = 0.600), gender (p = 0.492) and smoking status (p = 0.059) were not significantly correlated. In addition, no differences were found in NDRG1 expression according to the number (p = 0.111), size (p = 0.142) or pathological grade (p = 0.768) of tumours.

**Table 1:** Correlation between NDRG1 and clinical factors.

	Patients	NDRG1 expression		p value
		High (%)	Low (%)	
Total	100	35 (35.0)	65 (65.0)	
Age (years)				0.600
<60	20	8 (40.0)	12 (60.0)	
≥60	80	27 (33.8)	53 (66.2)	
Gender				0.492
Male	76	28 (36.8)	48 (63.2)	
Female	24	7 (29.2)	17 (70.8)	
Smoking				0.059
No	50	22 (44.0)	28 (56.0)	
Yes	50	13 (26.0)	37 (74.0)	
Multicentricity				0.111
No	52	22 (42.3)	30 (57.7)	
Yes	48	13 (27.1)	35 (72.9)	
Size (cm)				0.142
<3 cm	50	14 (28.0)	36 (72.0)	
≥3 cm	50	21 (42.0)	29 (58.0)	
Lymph node metastasis				0.034
Negative	94	30 (31.9)	64 (68.1)	
Positive	6	5 (83.3)	1 (16.7)	
TNM Stage				0.025
NMIBC	71	20 (28.2)	51 (71.8)	
MIBC	29	15 (51.7)	14 (48.3)	
Pathological grade				0.768
G1	33	10 (30.3)	23 (69.7)	
G2	44	16 (36.4)	28 (63.6)	
G3	23	9 (39.1)	14 (60.9)	

NMIBC: non-muscle-invasive bladder cancer; MIBC: muscle-invasive bladder cancer.

### High NDRG1 expression predicts poor prognosis in patients with bladder cancer

**Figure 3:**
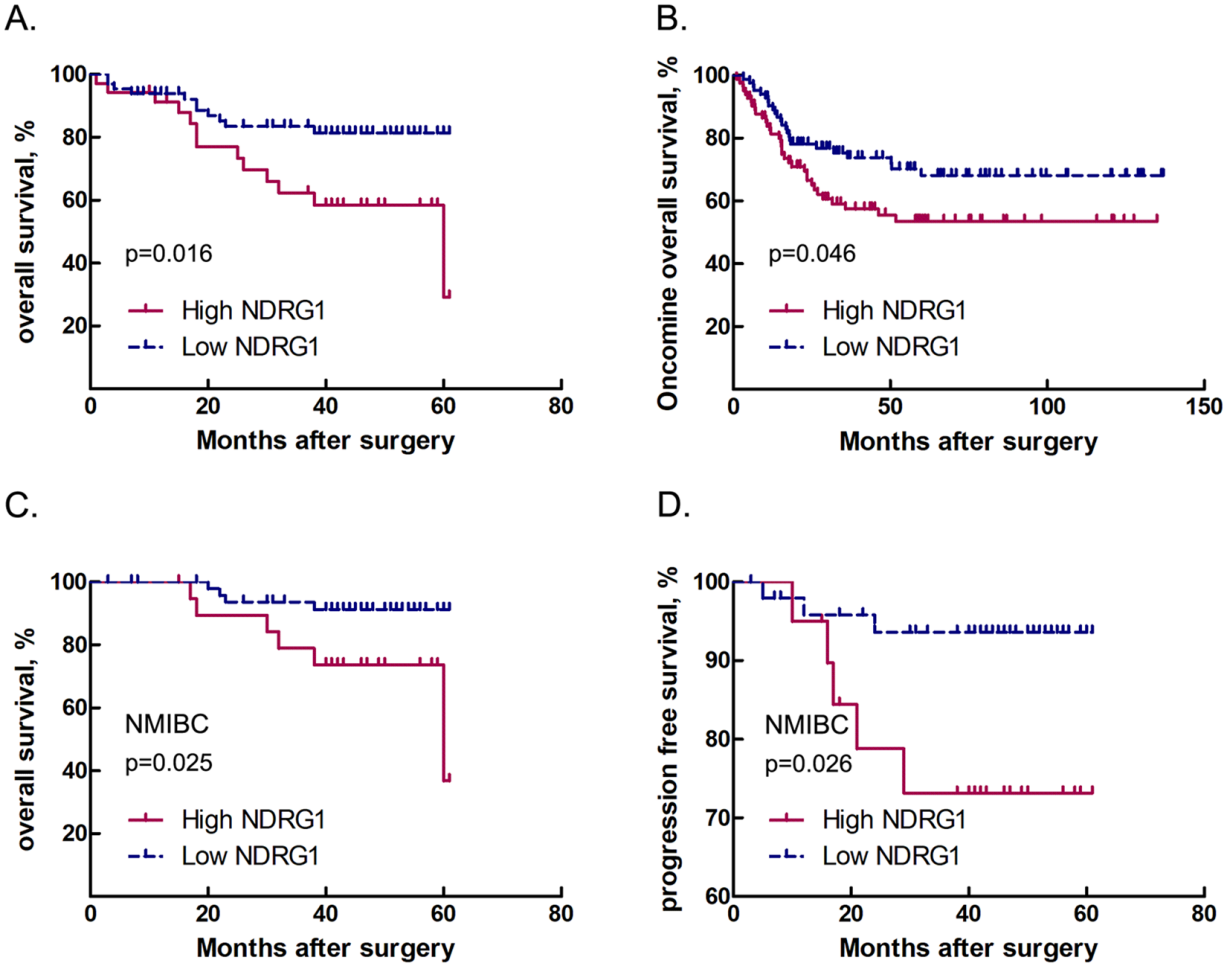
Correlations between NDRG1 expression and bladder cancer patient outcomes. (**A**) Overall survival of all patients with bladder cancer stratified by NDRG1 protein expression level; Kaplan-Meier method, log-rank test, p = 0.016. (**B**) Overall survival of bladder cancer patients from the Oncomine dataset stratified by NDRG1 mRNA expression level; Kaplan-Meier method, log-rank test, p = 0.046. (**C**) Overall survival (Kaplan-Meier curve, log-rank test, p = 0.025) and (**D**) progression-free survival of non-muscle-invasive bladder cancer (NMIBC) patients (Kaplan-Meier curve, log-rank test, p = 0.026).

To investigate the efficacy of NDRG1 as a prognostic biomarker, we further studied the correlation between NDRG1 expression and bladder cancer patient outcomes. The mean follow-up was 35 months (1–61 months), during which 46/100 experienced tumour recurrence, 18/46 experienced progression, and 24/100 died. Kaplan-Meier analysis showed that patients with high NDRG1 expression had significantly shorter survival times than those with low NDRG1 expression (mean survival time: 44 months vs. 53 months, p = 0.016) (Fig. 3A). In addition, we analysed 188 cases from the Oncomine database^23^ to correlate NDRG1 mRNA expression with overall survival. The result of this analysis was consistent with our data (p = 0.046) (Fig. 3B). Furthermore, among NMIBC patients, the high NDRG1 expression group had significantly shorter survival times than the low NDRG1 expression group (p = 0.025) (Fig. 3C), but the prognosis of these two groups of MIBC patients was not different (p = 0.612) (Fig. S1). High NDRG1 expression in NMIBC patients was correlated with a significantly higher risk of progression to MIBC (p = 0.026) (Fig. 3D). Moreover, multivariate Cox regression analysis showed that the level of NDRG1 expression was an independent prognostic factor in bladder cancer patients (B = 1.070; p = 0.012; RR (95% CI), 2.916 (1.264–6.724)).

### Expression of NDRG1 in bladder cancer cell lines

**Figure 4:**
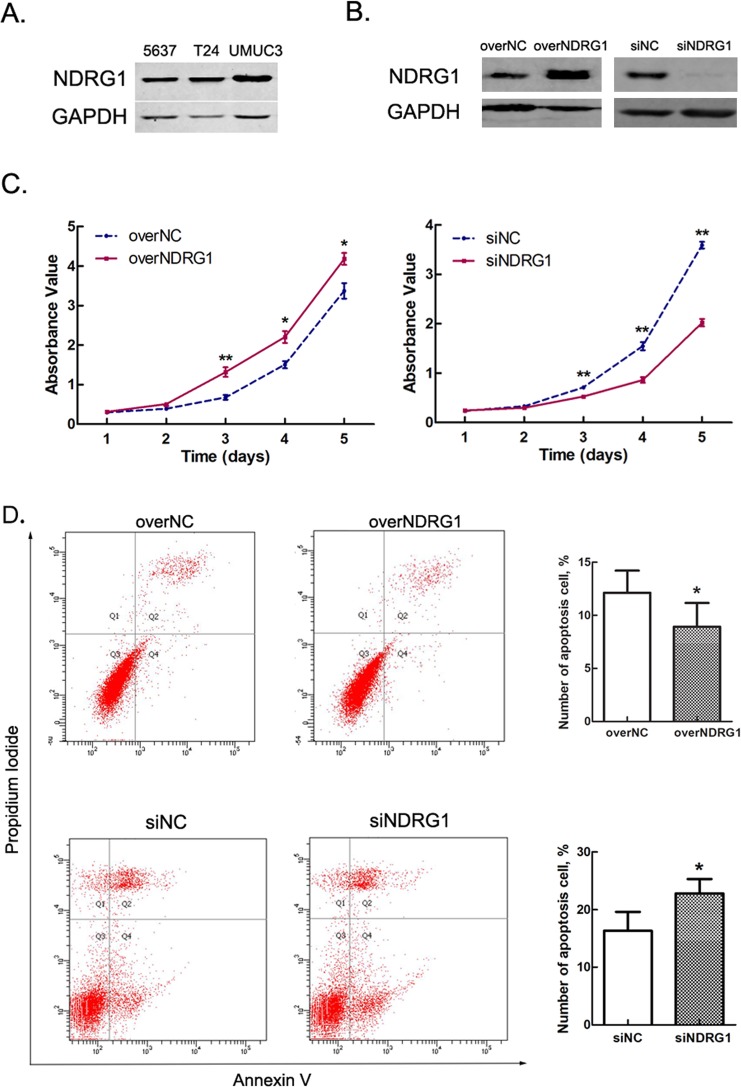
NDRG1 promotes the vitality of bladder cancer cells. (**A**) Expression of NDRG1 in various bladder cancer cell lines. (**B**) Expression of NDRG1 in 5637 cells after transfection with NDRG1 overexpression plasmid or siRNAs. (**C**) High NDRG1 expression promotes the proliferation of 5637 cells; n = 3, *p < 0.05, **p < 0.01. (**D**) High NDRG1 expression suppresses cell apoptosis; n = 3, *p < 0.05.

We chose 5637, T24 and UMUC3 cells for NDRG1 expression analysis. Western blot analysis showed the lowest expression of NDRG1 in 5637 cells, followed by T24 cells, and the highest expression of NDRG1 in UMUC3 cells (Fig. 4A and S2). Since UMUC3 cells are double-negative for E-cad and N-cad expression^24^, we selected 5637 and T24 cells for further study.

### NDRG1 affects bladder cancer cell viability by promoting proliferation and suppressing apoptosis

To clarify the role of NDRG1 in the growth of bladder cancer cells, 5637 cells treated with pcDNA3.1-NDRG1 or NDRG1 siRNAs were examined by a CCK-8 assay and an Annexin V-FITC/PI kit (Fig. 4). After NDRG1 overexpression, a significant increase in cell numbers was seen on day 3 (p = 0.009), day 4 (p = 0.017) and day 5 (p = 0.031), but no significant differences were seen on day 1 and day 2 (Fig. 4C). In addition, after NDRG1 knockdown, cell numbers were markedly decreased on day 3 (p = 0.001), day 4 (p = 0.002) and day 5 (p = 0.000) (Fig. 4C). The flow cytometry results showed that the number of apoptotic cells decreased (p = 0.015) after NDRG1 overexpression and increased (p = 0.028) after NDRG1 knockdown (Fig. 4D). However, the cell cycle assay did not reveal any appreciable change.

### NDRG1 enhances the motility of bladder cancer cells

**Figure 5:**
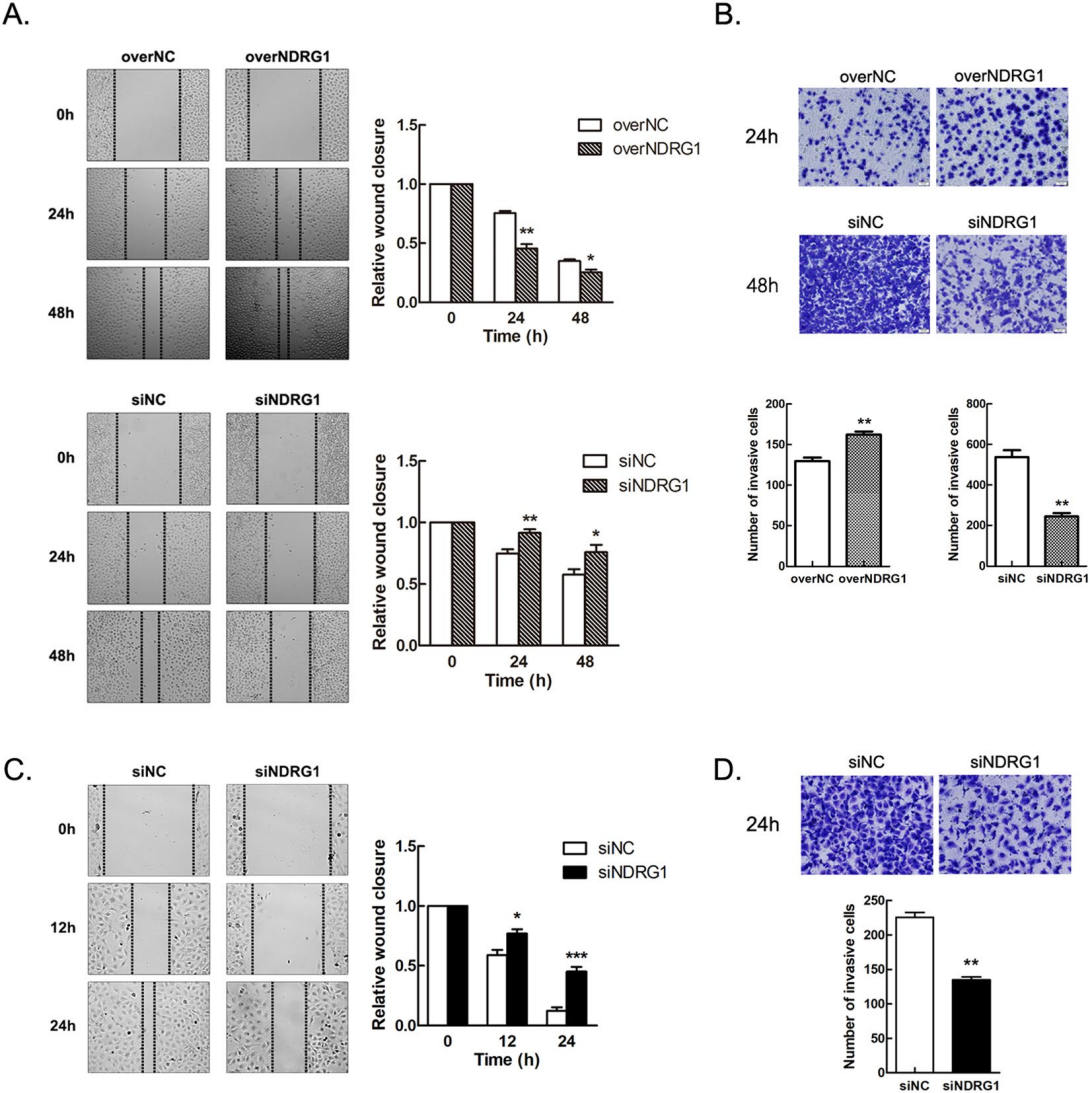
NDRG1 enhances the motility of bladder cancer cells. (**A**) After transfection, upregulated expression of NDRG1 promotes the migration of 5637 cells, and downregulated expression of NDRG1 suppresses the migration of 5637 cells and (**C**) T24 cells; n = 5, *p < 0.05, **p < 0.01, ***p < 0.0001. (**B**) NDRG1 expression affects the invasive ability of 5637 cells and (**D**) T24 cells; n = 5, **p < 0.01.

The effects of NDRG1 on cell motility were assessed with wound healing and Transwell assays. NDRG1-overexpressing 5637 cells exhibited a notably improved migration ability than the corresponding control cells (24 h, p = 0.002; 48 h, p = 0.029) (Fig. 5A). In addition, NDRG1 knockdown in 5637 cells inhibited migration (24 h, p = 0.006; 48 h, p = 0.040) (Fig. 5A). Furthermore, we validated these findings in T24 cells, which exhibit higher NDRG1 protein expression and cell viability than 5637 cells. T24 cells were transfected with NDRG1 siRNAs for 48 h, and a scratch wound was then made. For the same amount of healing time, the relative distance between the cells with low NDRG1 protein expression was greater than that between the control cells (12 h, p = 0.0111; 24 h, p = 0.000062) (Fig. 5C). Additionally, we stained the Transwell filters with crystal violet after cell culture (48 h for 5637 cells and 24 h for T24 cells) and found that NDRG1 noticeably affected the invasion of bladder cancer cells *in vitro* (p < 0.01) (Fig. 5B,D). After 24 h of transfection, the average number of invaded 5637 cells with NDRG1 overexpression was 162 (vs. control cells: 129). After 48 h of transfection, the average number of invaded 5637 cells with NDRG1 knockdown was 244 (vs. control cells: 537). Similarly, after 24 h of transfection, the average number of invaded T24 cells with NDRG1 knockdown was 135 (vs. control cells: 225).

### NDRG1 facilitates the process of EMT in bladder cancer cells

**Figure 6:**
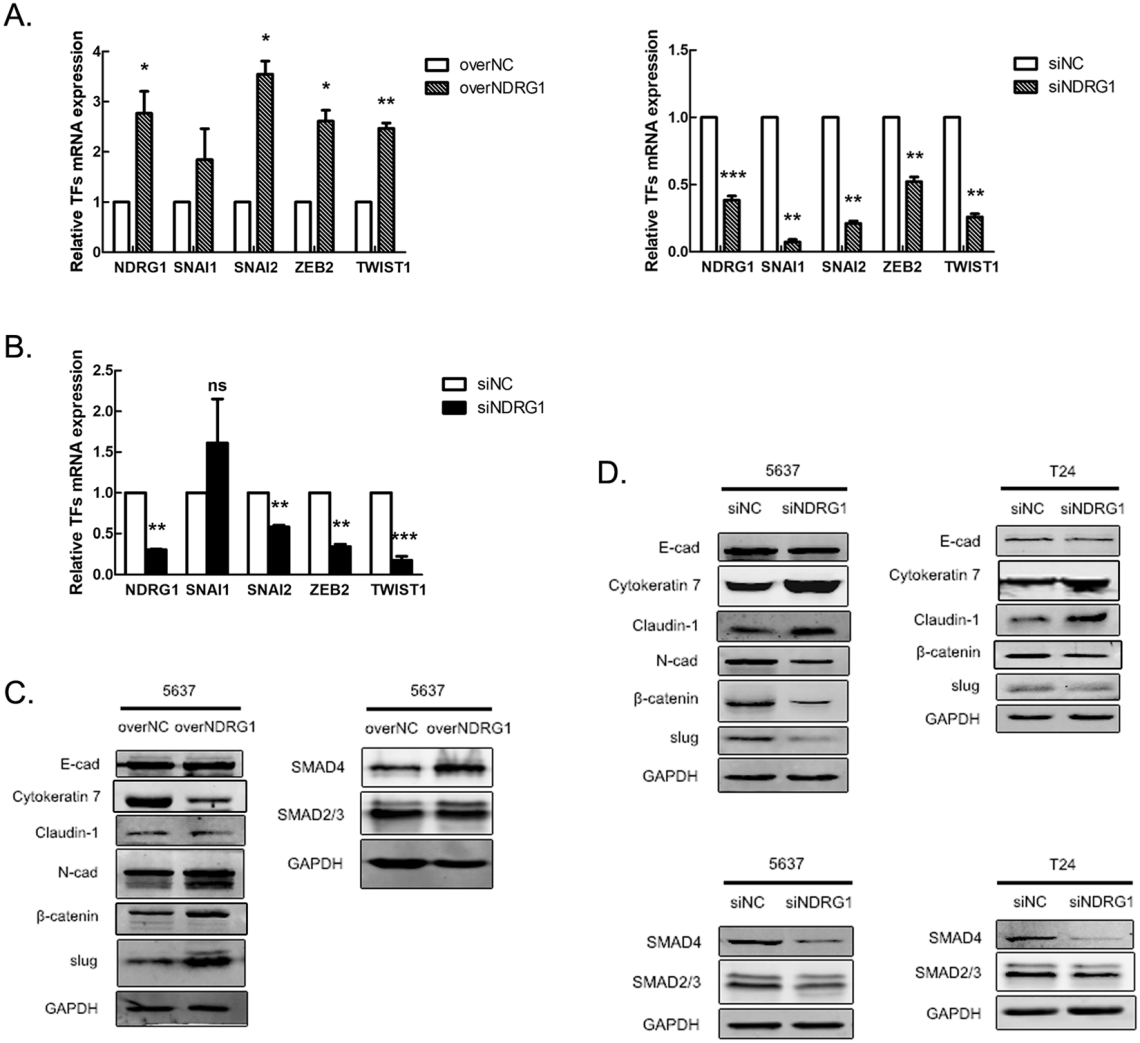
Changes in the expression of NDRG1 affect the EMT process of bladder cancer cells. The mRNA expression of EMT-related transcription factors (TFs) after the modulation of NDRG1 expression in (**A**) 5637 cells and (**B**) T24 cells; n = 3, *p < 0.05, **p < 0.01, ***p < 0.0001. Expression of EMT-related markers and the TGF-β pathway after the modulation of NDRG1 expression in (**C**) 5637 cells and (**D**) T24 cells; n = 3. EMT: epithelial-mesenchymal transition. TGF-β: transforming growth factor-β.

To further understand the mechanism by which NDRG1 mediates the development of bladder cancer, we assessed the expression levels of EMT-related markers after modulating NDRG1 expression in 5637 cells and T24 cells. Real-time PCR showed that after NDRG1 overexpression, the mRNA expression of EMT-related transcription factors (TFs), including SNAI2, ZEB2, and TWIST1, was significantly increased (p < 0.01), but the same change in only SNAI2 (slug) was found by western blot analysis (Fig. 6A,C). After NDRG1 knockdown, the expression of SNAI2, ZEB2, and TWIST1 was significantly decreased (p < 0.01) in both 5637 cells and T24 cells (Fig. 6A,B). Additionally, after NDRG1 overexpression, the protein expression of the epithelial markers Cytokeratin 7 and Claudin-1 was markedly decreased, whereas the expression of the mesenchymal markers N-cad and β-catenin was upregulated (Fig. 6C). Moreover, NDRG1 knockdown significantly increased the expression of Cytokeratin 7 and Claudin-1 and decreased the expression of β-catenin, slug, and N-cad (Fig. 6D). However, the expression of E-cad did not change appreciably in 5637 cells and T24 cells. We also investigated the expression of key molecules in the TGF-β pathway, one of the most important pathways for modulating EMT. Changes in NDRG1 expression mainly affected the expression of SMAD4, which then similarly changed the expression of SMAD4 (Fig. 6C,D).

### Urine NDRG1 protein levels could distinguish bladder cancer patients from healthy controls

**Figure 7:**
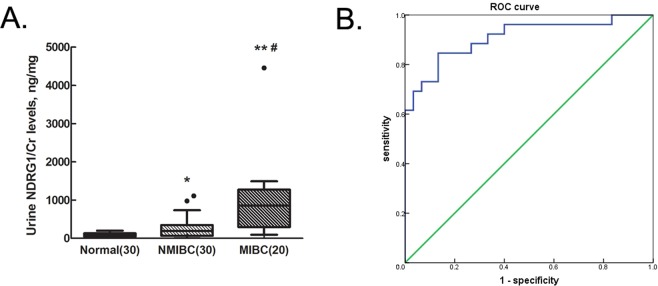
The diagnostic value of urine NDRG1 levels in bladder cancer patients. (**A**) The level of urine NDRG1 was measured in patients with NMIBC and MIBC and in healthy controls (healthy control vs. NMIBC: *p < 0.05; healthy control vs. MIBC: **p < 0.01; NMIBC vs. MIBC: ^#^p < 0.05). NMIBC: non-muscle-invasive bladder cancer, MIBC: muscle-invasive bladder cancer. (**B**) ROC analysis using NDRG1 to distinguish bladder cancer patients from healthy controls. ROC: Receiver operating characteristic. The area under the curve of NDRG1 expression to diagnose bladder cancer was 0.909 (95% CI, 0.829–0.989).

Considering that urine is the ideal body fluid to test for bladder cancer, we determined whether NDRG1 could be detected in patients’ urine by enzyme-linked immunosorbent assay (ELISA). The median levels of NDRG1/creatinine (Cr) in urine were 176.1 ± 270.0 ng/mg in patients with NMIBC and 779.1 ± 1019.6 ng/mg in patients with MIBC, both of which were significantly higher than that in urine from healthy controls (69.8 ± 86.4 ng/mg; pNMIBC = 0.033, pMIBC = 0.0005) (Fig. 7A). The level of NDRG1 was also higher in urine from patients with MIBC than in urine from patients with NMIBC (p = 0.029) (Fig. 7A). Next, the receiver operating characteristic (ROC) curve based on the ELISA results was plotted to evaluate the potential of urinary NDRG1 as a non-invasive biomarker for the diagnosis of bladder cancer. The ROC curve demonstrated that the protein level of NDRG1 could distinguish bladder cancer patients from healthy controls (Fig. 7B). The optimum cutoff value for the diagnosis of bladder cancer patients was 183.2 ng/mg. The area under the curve (AUC) of NDRG1 expression to diagnose bladder cancer was 0.909 (95% CI, 0.829–0.989), with a sensitivity of 84.6% and a specificity of 86.7%.

## Discussion

Although bladder cancer can usually be cured by surgical and nonsurgical therapies, recurrence and metastasis remain the major challenges in clinical practice and are the most common cause of death in patients^2^. Based on this observation, accurate risk stratification and intensive surveillance become even more critical to improve outcomes. The present risk assessment system for NMIBC, including tumour multicentricity, size, TNM stage, and pathological grade, was established for roughly predicting patients’ outcomes, but its accuracy remains to be improved^25^. Therefore, effective biomarkers are needed.

NDRG1 was historically identified as a protein involved in the differentiation of epithelial cells^4,26^. Previous studies showed that NDRG1 could be a tumour suppressor in colon, prostate and pancreatic cancer^14,27,28^. However, contrasting results were found in other types of cancers. For example, the NDRG1 protein was significantly overexpressed in human hepatocellular carcinoma (HCC) samples relative to its expression in non-tumour liver samples or cirrhotic and benign liver lesion samples, and this overexpression was associated with vascular invasion and poor survival^12^. Furthermore, NDRG1 expression was correlated with metastasis and recurrence in HCC patients after liver transplantation, and positive NDRG1 expression could be important, indicating a poor prognosis^29^. In cervical adenocarcinoma, high NDRG1 expression was directly associated with shorter progression-free survival and overall survival^30^. We demonstrated here for the first time that the NDRG1 mRNA and protein expression levels are significantly upregulated in bladder cancer tissues. The level of NDRG1 expression was an independent prognostic factor. High NDRG1 expression was associated with poor overall survival and increased risk of progression to MIBC in NMIBC patients. A previous study showed that the localization of NDRG1 expression, especially in the nucleus, could predict tumour angiogenesis and poor prognosis in non-small cell lung cancer (NSCLC)^31^. However, the localization of NDRG1 was not a significant indicator of prognosis in our study.

Although NDRG1 mainly exists as an intracellular protein, it can be stably detected extracellularly. In NSCLC, NDRG1 was found to be detectable as a distinguishing marker in the serum of patients by ELISA^16^, suggesting that it could be a serum/urine biomarker. Tumour biomarkers for bladder cancer can be categorized by source into three types: tissue, serum, and urine. Urine biomarkers are especially attractive because urine is easy to collect and contains considerable bioinformation about the primary tumour site in the bladder^32^. Currently, some markers, such as NMP-22 and BTA, have been approved by the U.S. Food and Drug Administration (FDA) for the diagnosis or surveillance of bladder cancer^33^. However, none have been accepted in routine practice or by clinical guidelines. A meta-analysis showed that the sensitivity of the quantitative NMP-22 test was 0.69 (95% CI, 0.62–0.75), and the specificity was 0.77 (95% CI, 0.70–0.83) (19 studies)^33^. The results of the qualitative NMP-22 test were not as accurate, with a sensitivity of only 0.58 (95% CI, 0.39–0.75) and a specificity of 0.88 (95% CI, 0.78–0.94)^33^. Interestingly, a recent study showed that NDRG2, which belongs to the NDRG family with NDRG1, might be a urine-based biomarker for bladder cancer^34^. The results of a western blot analysis showed that NDRG2 expression was lower in the urine of bladder cancer patients than in the urine of healthy controls^34^. ROC analysis showed an AUC of 0.888 (95% CI, 0.845–0.930), a sensitivity of 85.5%, and a specificity of 81.4%^34^. We found that urine NDRG1 could be a promising biomarker for the diagnosis of bladder cancer, with an AUC of 0.909 (95% CI, 0.829–0.989), an acceptable sensitivity of 84.6% and a specificity of 86.7%. The NDRG2 protein is expressed at high levels in skeletal muscle tissue and at low levels in tumour tissue^35^. Although the amino acid sequence of the NDRG2 protein has 57–65% homology with that of NDRG1, these proteins play different roles in the cell^35^. Considering the indicative role of these biomarkers, we believe that identifying upregulated NDRG1 protein levels may be more meaningful than identifying downregulated NDRG2 protein levels and we believe that ELISA is more suitable than western blotting for measuring the protein level in the urine.

In addition, our *in vitro* experimental data showed that NDRG1-modulated bladder cancer cells grew faster via the promotion of proliferation and inhibition of apoptosis. Moreover, cell motility increased with NDRG1 overexpression but decreased with NDRG1 knockdown. Taken together, these results showed that NDRG1 mediated the invasive bladder cancer phenotype. EMT is a crucial initiator of invasiveness for epithelial-derived tumours, featuring a switching off of epithelial marker expression and a switching on of mesenchymal marker expression^17^. The classic characteristics of EMT are the loss of cell-cell junctions and the reorganization of the cytoskeleton^17^. Recently, EMT-related TFs, mainly of the SNAI, ZEB and TWIST families, which are called the executors of the EMT process, have been reported to play important roles in all stages of cancer progression^17,36^. We assessed the expression of SNAI1 (snail), SNAI2 (slug), ZEB1, ZEB2, TWIST1, and TWIST2 by real-time PCR and western blot analyses and found that the mRNA expression of SNAI1, SNAI2, ZEB2, and TWIST1 was significantly changed after the modulation of NDRG1 expression but that ZEB1 and TWIST2 expression was not detected in 5637 cells and T24 cells. However, the protein expression of only SNAI2 (slug) was detected, suggesting that slug might be the main TF affecting EMT in bladder cancer. Slug, as well as snail and twist, could induce the loss of E-cad expression and the upregulation of N-cad expression in bladder cancer cells^37,38^. Furthermore, NDRG1 mainly affected N-cad expression instead of E-cad expression in our study. Currently, the role of N-cad in EMT seems well established, facilitating cell motility and migration as a prelude to invasion and metastasis and inducing or accompanying significant changes in growth factor signalling^39^. Cytokeratin 7, which is considered an epithelial marker for EMT, was selected for analysis because it is expressed in all epithelial cells of normal urothelial and NMIBC tissue but not in stromal cells^40–42^. Claudin-1, one of the proteins composing tight junctions, is also considered an epithelial marker. The expression of these two proteins was noticeably influenced by NDRG1 in our study, indicating that NDRG1 could affect the process of EMT. TGF-β signalling is one of the best-known signalling cascades in EMT, acting canonically through smad proteins to induce EMT-related TFs^43^. Moreover, TGF-β/smad signalling downregulates the expression of ZO-1, Claudin-1, and cytokeratins, followed by the degradation of tight junctions and the upregulation of N-cad expression^43,44^. In the classical pathway, SMAD4, as the common partner smad (co-SMAD), forms complexes with p-SMAD2/p-SMAD3 and then translocates into the nucleus and regulates the transcription of target genes through interactions with other DNA-binding TFs, such as snail/slug^45^. We found that SMAD4 expression significantly increased with NDRG1 overexpression but decreased with NDRG1 knockdown, primarily causing the alteration of Cytokeratin 7, Claudin-1 and N-cad expression, suggesting that NDRG1 might influence the process of EMT through modulating the expression of SMAD4 and slug in bladder cancer. The Wnt/β-catenin pathway is another classical signalling pathway regulating EMT^44^. NDRG1 was reported to be a substrate for GSK-3β and can directly interact with p-GSK-3β^46^. When NDRG1 was highly expressed, it competed with β-catenin for binding to p-GSK-3β, which helped β-catenin escape the degradation process and translocate into the nucleus to activate the expression of target genes and promote the EMT process^46^. The results of our study confirmed that when NDRG1 was overexpressed, the expression of β-catenin was significantly increased.

In conclusion, to our knowledge, our study provided the first evidence that high expression of NDRG1 in bladder cancer patients is associated with advanced tumour stage and poor outcomes and could be a diagnostic and prognostic biomarker. In addition, NDRG1 might promote tumour development via the EMT pathway by modulating SMAD4/slug expression.

## Materials and Methods

### Patients

A total of 100 patients with primary bladder cancer, including NMIBC (71 patients) and MIBC (29 patients), from Friendship Hospital Affiliated to Capital Medical University were enrolled in this study. None of the patients had a history of any other types of tumour and received preoperative chemotherapy, radiotherapy or other cancer-related treatments. Pathological sections for NDRG1 expression analysis and clinical factors—namely, age, sex, tumour number and size, clinical TNM stage and pathological grade—were collected from the patients, and all patients were followed up for five years after surgery. The outcomes, including recurrence, progression and death, were recorded.

15 pairs of freshly frozen bladder carcinoma and corresponding tumour-free tissues were collected from the bladder cancer patients for evaluation of NDRG1 mRNA and protein expression.

### Urine samples

A total of 30 urine samples from patients with NMIBC and 20 urine samples from patients with MIBC were collected into sterile containers before initial treatment to assess the levels of NDRG1 and Cr, and 30 urine samples from healthy volunteers were used as the controls. All samples were centrifuged at 4 °C and 1000 × g for 10 min within 4 h of collection. The supernatant was stored at −80 °C until analysis. The concentration of Cr was measured by a Beckman Coulter AU5800 (Beckman Coulter, Inc., US) as the internal control.

### IHC and immunoscoring

Pathological sections were incubated with anti-NDRG1 antibody (1:600; Abcam, UK) at 4 °C overnight, followed by incubation for 30 min at 25 °C with horseradish peroxidase (HRP)-conjugated secondary antibody (ZSGB-BIO, China). Immunostaining was performed using a DAB Detection Kit (Polymer) (ZSGB-BIO, China), and counterstaining was performed with haematoxylin. NDRG1 expression was quantified using the German semi-quantitative scoring system by two independent analysts. The final immunostaining score of each section was determined by multiplying the intensity score (0, no staining; 1, weak staining; 2, moderate staining; 3, strong staining) and the percentage score of stained tumour cells (<10% = 0, 10–25% = 1, 26–50% = 2, 51–75% = 3, 76–100% = 4). Sections with scores of 1–4 were considered to exhibit low expression, and those with scores of 5–12 were considered to exhibit high expression.

### Cell culture and transfection

The human bladder cancer cell lines 5637, T24 and UMUC3 were obtained from ATCC (Rockville, US) and cultured in RPMI 1640 medium (HyClone, US) supplemented with 10% foetal bovine serum, 100 U/ml penicillin and 100 μg/ml streptomycin in an incubator with 5% CO_2_ at 37 °C.

The pcDNA3.1-NDRG1 (GenBank number: NM_001135242.1) plasmid was designed and NDRG1 SignalSilence siRNAs (siRNA I: #6245 and siRNA II: #6257; Cell Signaling Technology, US) were purchased to regulate NDRG1 expression. The plasmid and siRNAs were then transiently transfected into bladder cancer cells by Attractene Transfection Reagent or HiPerFect Transfection Reagent (QIAGEN, Germany). The two of NDRG1 siRNAs were mixed before use, and pcDNA3.1 and control siRNA (#6568, Cell Signaling Technology, US) were transfected as the controls. Two days after transfection, cells were harvested.

### RNA extraction and real-time PCR analysis

Total RNA was extracted with Trizol (Invitrogen, US), and cDNA was synthesized by a RevertAid First Strand cDNA Synthesis Kit (Thermo Scientific, US) according to the manufacturer’s protocol. The mRNA expression levels of NDRG1 and EMT-related TFs were assessed by real-time PCR. GAPDH was used as the internal control. Real-time PCR was performed using the Power SYBR Green (Applied Biosystems, US) dye detection method on an Applied Biosystems 7500 Real-Time PCR system under the default conditions: 95 °C for 10 min, followed by 40 cycles of 95 °C for 15 s and 60 °C for 60 s. Comparative Ct values were calculated as 2^−ΔΔCt^ for transcript quantification. The primer sequences used are shown in Table S1.

### Protein extraction and western blot analysis

Total protein was harvested in RIPA buffer. Protein extraction and western blotting were performed as described previously^47^. Anti-NDRG1 (#9485 1:1000, Cell Signaling Technology, US) and anti-GAPDH (3 µg/ml, MBL Co., Ltd, Japan) primary antibodies were used. Other antibodies are shown in Table S2.

### Cell proliferation, cell cycle and apoptosis assays

After transfection, 5637 cells were seeded into 96-well plates at a density of 6 × 10^3^ cells per well. Cell growth was measured for five days with a cell counting kit (ZP328; ZOMANBIO, China) according to the manufacturer’s instructions. Absorbance was measured at 450 nm. The cell cycle progression and apoptosis of 5637 cells after transfection were measured on a FACSCanto II flow cytometer (Becton Dickinson, US) by an Annexin V-FITC/PI Apoptosis Detection Kit (ZP327-1; ZOMANBIO, China), following the manufacturer’s instructions.

### *In vitro* migration and invasion assays

Cell migration was assessed by a wound healing assay. After transfection, bladder cells were cultured to form a confluent cell monolayer, and a 10-μl plastic pipette tip was then used to scratch the monolayer. At the indicated time points, the percentage of the cell-free area compared with that at time zero was measured. Cell invasion assays were carried out using an 8-μm Transwell chamber coated with Matrigel (Corning, US), according to the manufacturer’s protocol.

### ELISA

The urine NDRG1 assay was performed using a commercially available ELISA kit (OKEH02359, Aviva Systems Biology, US) according to the manufacturer’s protocol. The NDRG1 ELISA kit is based on standard sandwich ELISA technology. The concentration of NDRG1 was calculated using a four-parameter logistic curve.

### Gene expression profile analysis

Datasets from Oncomine (https://www.oncomine.org) were extracted and used to validate the expression pattern of NDRG1 in bladder cancer, following strict human subjects protection guidelines.

### Statistics

Each experiment was repeated in triplicate. SPSS ver.20.0 (SPSS Inc., US) was used for all analyses, and the significance level was set at p < 0.05. A chi-squared test was used for categorical variables, and Student’s t-test was used for continuous variables. Survival analysis was performed using the Kaplan-Meier method and log-rank test. Multivariate analyses were performed using a Cox proportional hazards model to identify prognostic factors. ROC analysis and Youden’s index (Youden’s index = sensitivity + specificity − 1) were used to identify the cutoff, sensitivity, specificity and AUC values.

### Ethical approval

The study was approved by the Ethics Committee of Friendship Hospital Affiliated to Capital Medical University. All procedures performed in studies involving human participants were in accordance with the ethical standards of the institutional and/or national research committee and with the 1964 Helsinki declaration and its later amendments or comparable ethical standards. Informed consent was obtained from all participants enrolled in the study.

## Supplementary information

supplementary material
