# Supplementary material for: Upregulation of NDRG1 predicts poor outcome and facilitates disease progression by influencing the EMT process in bladder cancer
Source: Sci Rep. 2019 Mar 26;9:5166. doi: 10.1038/s41598-019-41660-w (PMC6435802; doi:10.1038/s41598-019-41660-w)

**Upregulation of NDRG1 predicts poor outcome and facilitates disease progression by influencing the EMT process in bladder cancer.**

Aiwei Li^1^, Xi Zhu^2^, Chanjuan Wang^1^, Shuo Yang^1^, Yan Qiao^1^, Rui Qiao^1^, Jie Zhang^1^*

1 Department of Laboratory Medicine, Peking University Third Hospital, No.49 North Garden Road, Haidian District, Beijing 100191, China

2 Department of Urology, Friendship Hospital Affiliated to Capital Medical University, 95th Yong An Road, Xuan Wu District, Beijing 100050, China

***Correspondence:**

Pro. Dr. Jie Zhang, Department of Laboratory Medicine, Peking University Third Hospital, E-mail: [zhangjiebjmu@163.com](mailto:zhangjiebjmu@163.com), Phone: +86 10 82265719, Fax: +86 10 82265719

Table S1 The sequences of all primers listed in this study

| Target gene | Forward primer (5′–3′) | Reverse primer (5′ –3′) |
| --- | --- | --- |
| NDRG1 | CGCCAGCACATTGTGAATGAC | TTTGAGTTGCACTCCACCACG |
| SNAI1 | CCTCCCTGTCAGATGAGGAC | CCAGGCTGAGGTATTCCTTG |
| SNAI2 | GGGGAGAAGCCTTTTTCTTG | TCCTCATGTTTGTGCAGGAG |
| ZEB1 | AACCCAACTTGAACGTCACA | ATTACACCCAGACTGCGTCA |
| ZEB2 | CCAGAGGAAACAAGGATTTCAG | AGGCCTGACATGTAGTCTTGTG |
| TWIST1 | AGCTACGCCTTCTCCGTCT | TCCTTCTCTGGAAACAATGACA |
| TWIST2 | GAGCGACGAGATGGACAATAAGA | ATGCGCCACACGGAGAA |
| GAPDH | TGTTCCAATATGATTCCACCC | CTTCTCCATGGTGCGTGAAGA |

Table S2 The antibody of all protein listed in this study

| Target protein | Dilution | CAT. # | Manufacturer |
| --- | --- | --- | --- |
| NDRG1 | 1:1000 | 9485 | Cell Signaling Technology, US |
| E-cad | 1:500 | ab40772 | Abcam, US |
| N-cad | 1:500 | Ab76011 | Abcam, US |
| Cytokeratin 7 | 1:5000 | ab68459 | Abcam, US |
| Claudin-1 | 1:500 | 13255 | Cell Signaling Technology, US |
| β-catenin | 1:1000 | 8480 | Cell Signaling Technology, US |
| Slug | 1:250 | 9585 | Cell Signaling Technology, US |
| SMAD4 | 1:1000 | 38454 | Cell Signaling Technology, US |
| SMAD2/3 | 1:1000 | 8685 | Cell Signaling Technology, US |
| GAPDH | 3 µg/mL | M171-3 | MBL Co., Ltd, Japan |

Table S3 Patients’ characteristics of 15 paired bladder cancer tissues

|  | Patients |
| --- | --- |
|  |  |
| Total | n=15 |
| Age (years) |  |
| <60 | 3 |
| ≥60 | 12 |
| Gender |  |
| Male | 12 |
| Female | 3 |
| Smoking |  |
| No | 6 |
| Yes | 9 |
| Multicentricity |  |
| No | 10 |
| Yes | 5 |
| Size (cm) |  |
| <3cm | 5 |
| ≥3cm | 10 |
| Lymph node metastasis |  |
| Negative | 13 |
| Positive | 2 |
| TNM Stage |  |
| NMIBC | 4 |
| MIBC | 11 |
| Pathological grade |  |
| G1 | 2 |
| G2 | 7 |
| G3 | 6 |

NMIBC: non-muscle-invasive bladder cancer;

MIBC: muscle-invasive bladder cancer

Fig. S1


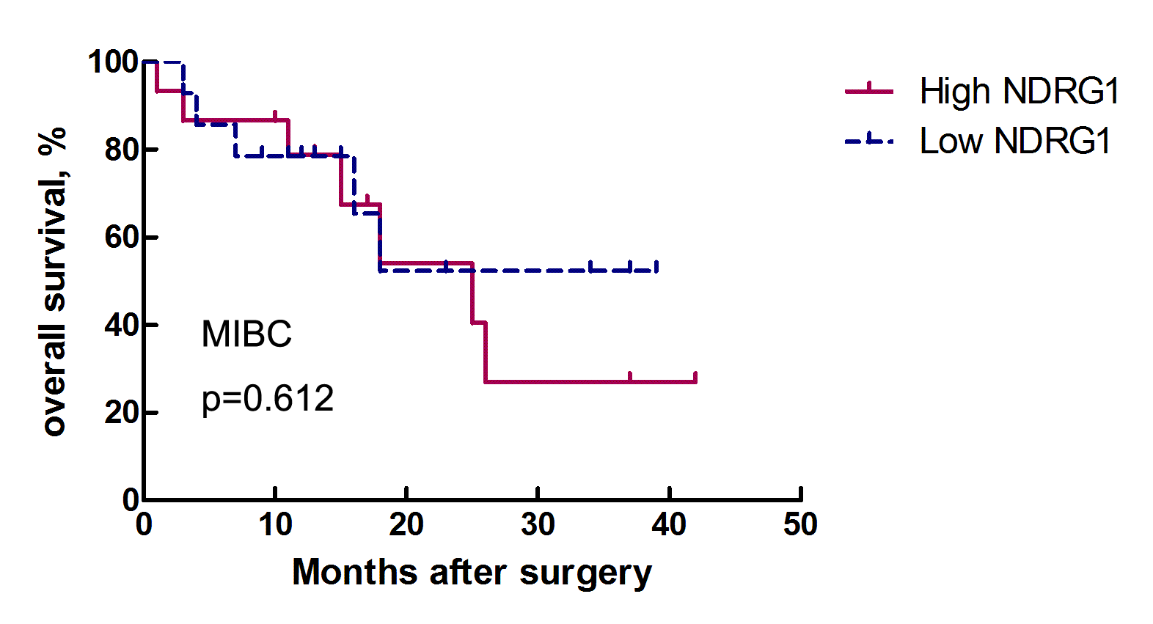


Overall survival of patients with muscle invasive bladder cancer (MIBC) stratified by NDRG1 protein expression level; Kaplan-Meier method, log-rank test, p=0.612.

Fig. S2





Expression of NDRG1 in various bladder cancer cell lines. n=3, **p<0.01, ***p<0.0001.

Fig. S3


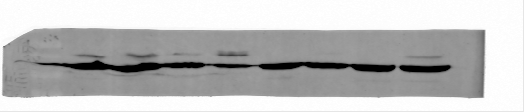


T24

siNC siNDRG1


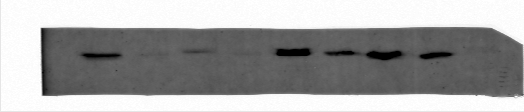


Expression of NDRG1 in T24 cells after transfection with NDRG1 siRNAs.

Figure 1C Original image

NDRG1
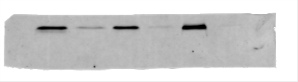


GAPDH
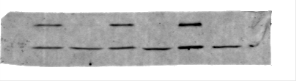


NDRG1
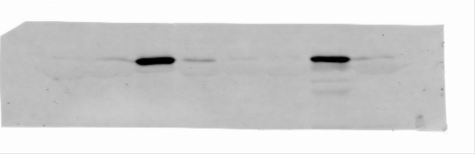


GAPDH
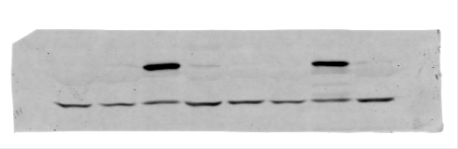


NDRG1


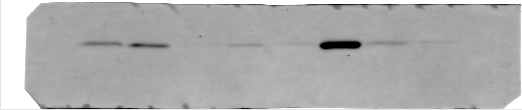


GAPDH
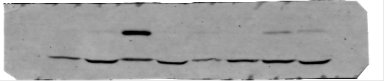


NDRG1


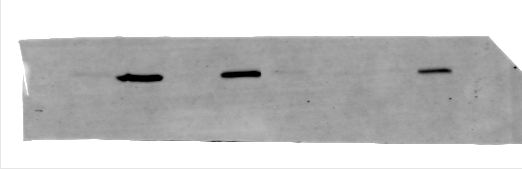


GAPDH


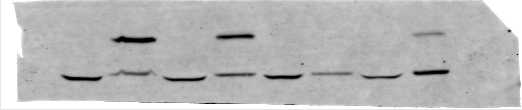


Figure 4A Original image

NDRG1


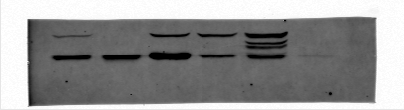


GAPDH


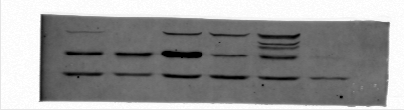


Figure 4B Original image

NDRG1 over
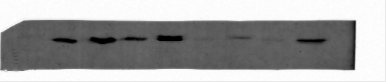


GAPDH


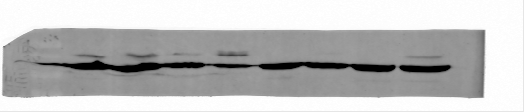


NDRG1 si



GAPDH


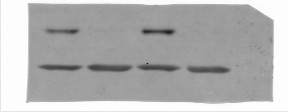


Figure 6C Original image

E-cad



GAPDH


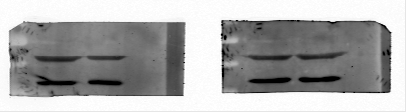


Cytokeratin 7


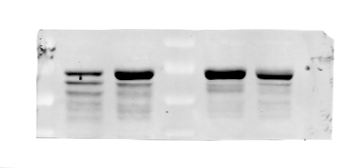


Claudin-1
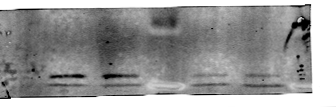


GAPDH
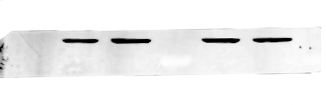


N-cad


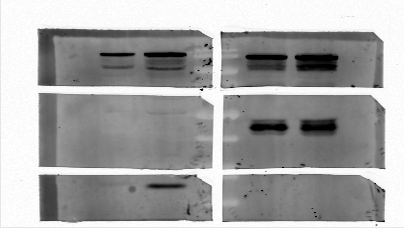


GAPDH


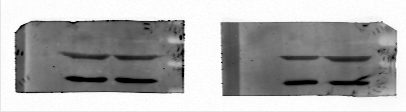


β-catenin


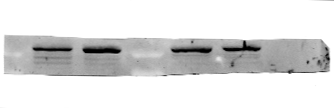


slug



SMAD4



GAPDH


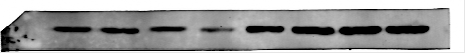


SMAD2/3


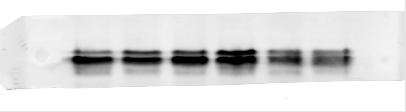


GAPDH


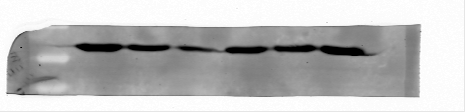


Figure 6D Original image

5637 cell

Cytokeratin 7
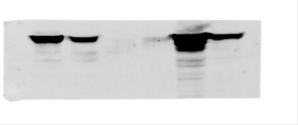


GAPDH



Claudin-1


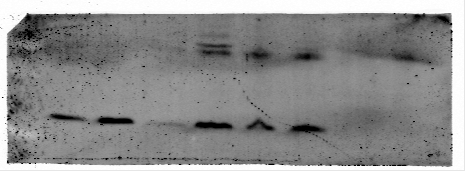


GAPDH


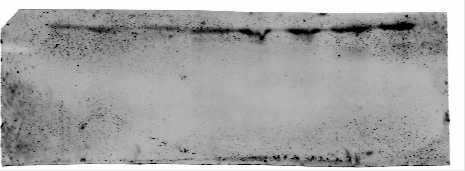


N-cad



slug



GAPDH



E-cad



β-catenin



GAPDH



SMAD4



GAPDH



SMAD2/3



GAPDH



T24 cell

E-cad


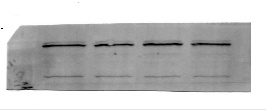


GAPDH


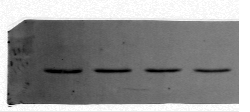


Cytokeratin 7



GAPDH



Claudin-1
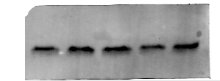


slug
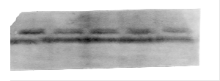


GAPDH
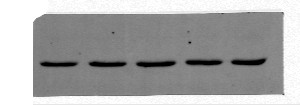


β-catenin


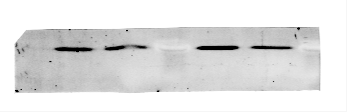


GAPDH


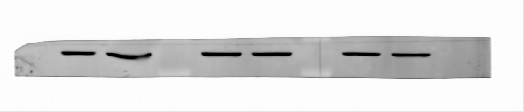


SMAD4


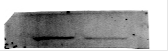


SMAD2/3


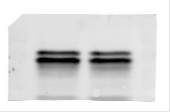


GAPDH


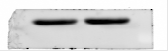

Supplement: Supplementary file 1 — supplementary material [file 41598_2019_41660_MOESM1_ESM.docx]
